# Supplementary material for: A framework for research into continental ancestry groups of the UK Biobank
Source: Hum Genomics. 2022 Jan 29;16:3. doi: 10.1186/s40246-022-00380-5 (PMC8800339; doi:10.1186/s40246-022-00380-5)
Supplement: Supplementary file 2 — Additional file 2. Figure S1. Continental ancestry PCA scree plots. Figure S2. K-means k selection with silhouette analysis. Figure S3. UK Biobank continental ancestry group PCs with K-means clusters. Figure S4. Population structure by UN defined geographic region. Figure S5. Population structure centers, as defined by UN geographic region. Figure S6. Population structure by country of birth in Africa by region. Figure S7. Population structure by country of birth in Europe by region. Figure S8. Population structure by country of birth in South Asia and East Asia. Figure S9. Population structure centers by country of birth. Figure S10. Population structure centers by country of birth. [file 40246_2022_380_MOESM2_ESM.docx]

# SUPPLEMENTARY FIGURES for: A framework for research into continental ancestry groups of the UK Biobank

By: Andrei-Emil Constantinescu, Ruth Mitchell, Borko Amulic, Jie Zheng, Caroline Bull, Nicholas J. Timpson, Emma E. Vincent, David A. Hughes

## Supplementary Figure 1: Continental ancestry PCA scree plots


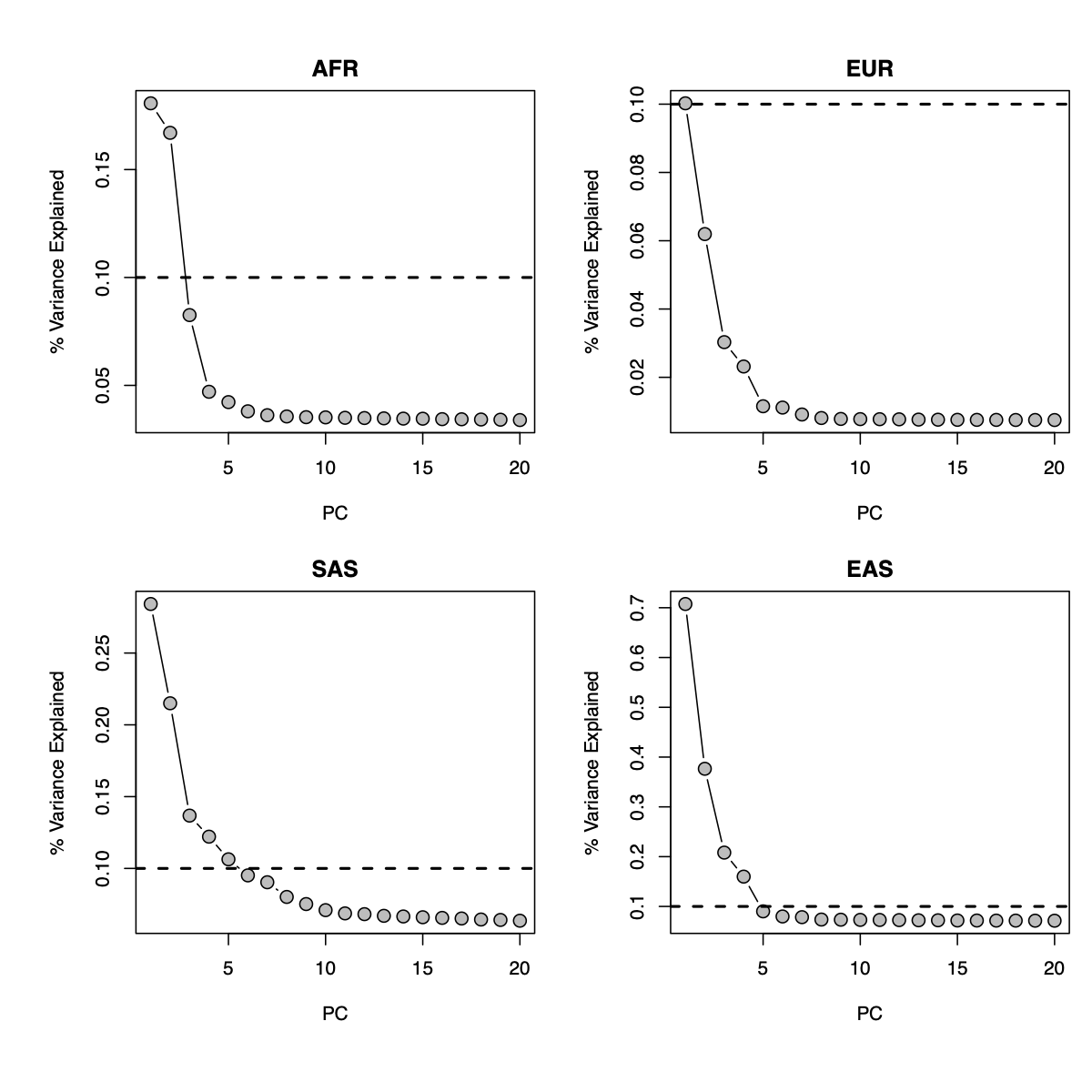


Legend: Scree plots illustrating the proportion of variation explained by each of the top 20 PCs, in each UKBB continental ancestry principal component analyses. The Scree plots were used to identify the number of top PCs to carry forward into the k-means clustering analysis. The continental ancestry supergroups are Africa (AFR), Europe (EUR), South Asia (SAS), and East Asia (EAS). The number of PCs selected as top PCs are AFR = 4, SAS = 5, EAS = 4, EUR = 5.

## Supplementary Figure 2: K-means k selection with silhouette analysis


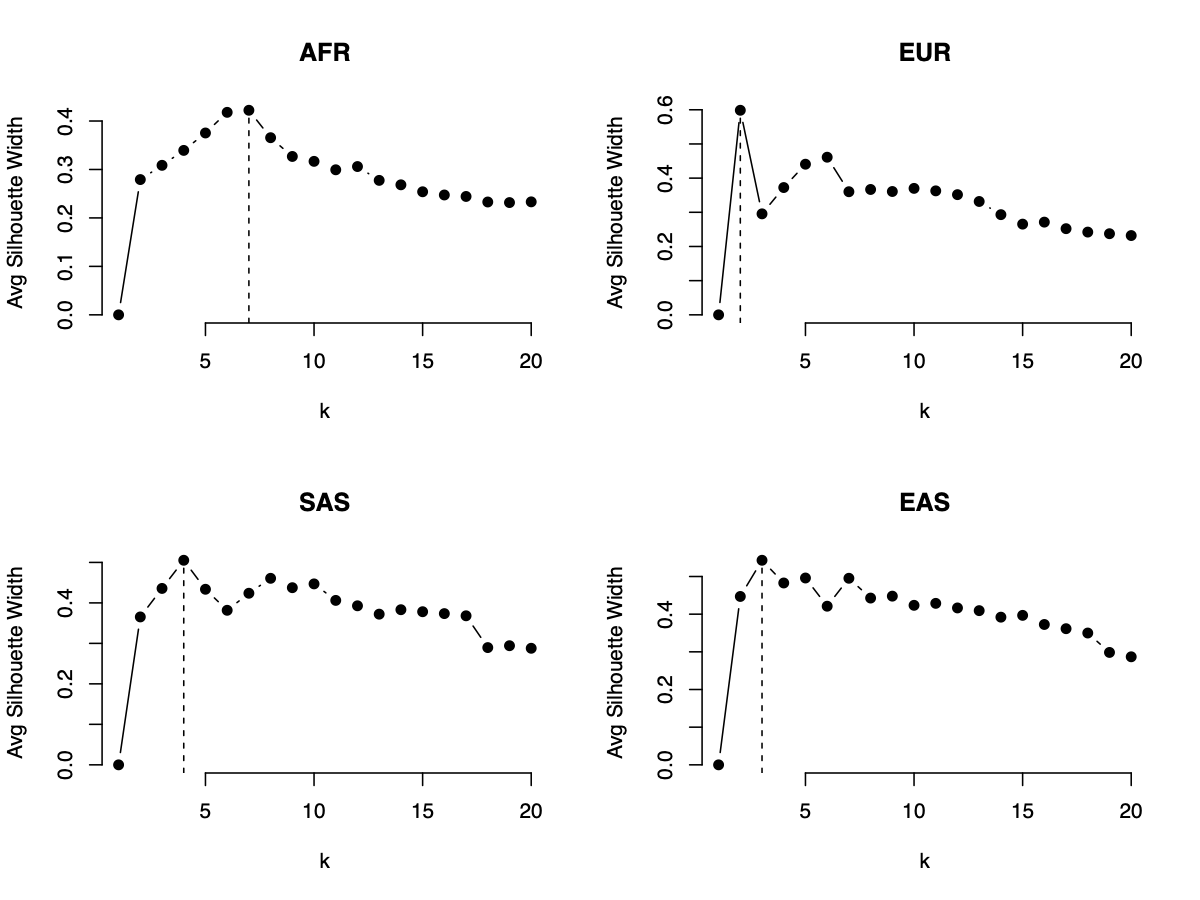


Legend: Selection of an optimum number of k clusters in the k-means analysis of the top PCs, by silhouette analysis. A silhouette plot for each UKBB continental supergroup (AFR) African, (EUR) European, (SAS) South Asian, and (EAS) East Asian is provided. The x-axis indicates the number of k clusters evaluated, and the y-axis provides an estimate of the average silhouette width (ASW). ASW is an estimation of cluster quality, or intra- and inter- cluster distances derived from a partitioning around medoids (PAM). The optimum number of k clusters in each UKBB continental supergroup were identified as AFR = 7, EUR = 2, SAS = 4, and EAS = 3.

## Supplementary Figure 3: UKBB continental ancestry group PCs with K-means clusters

##
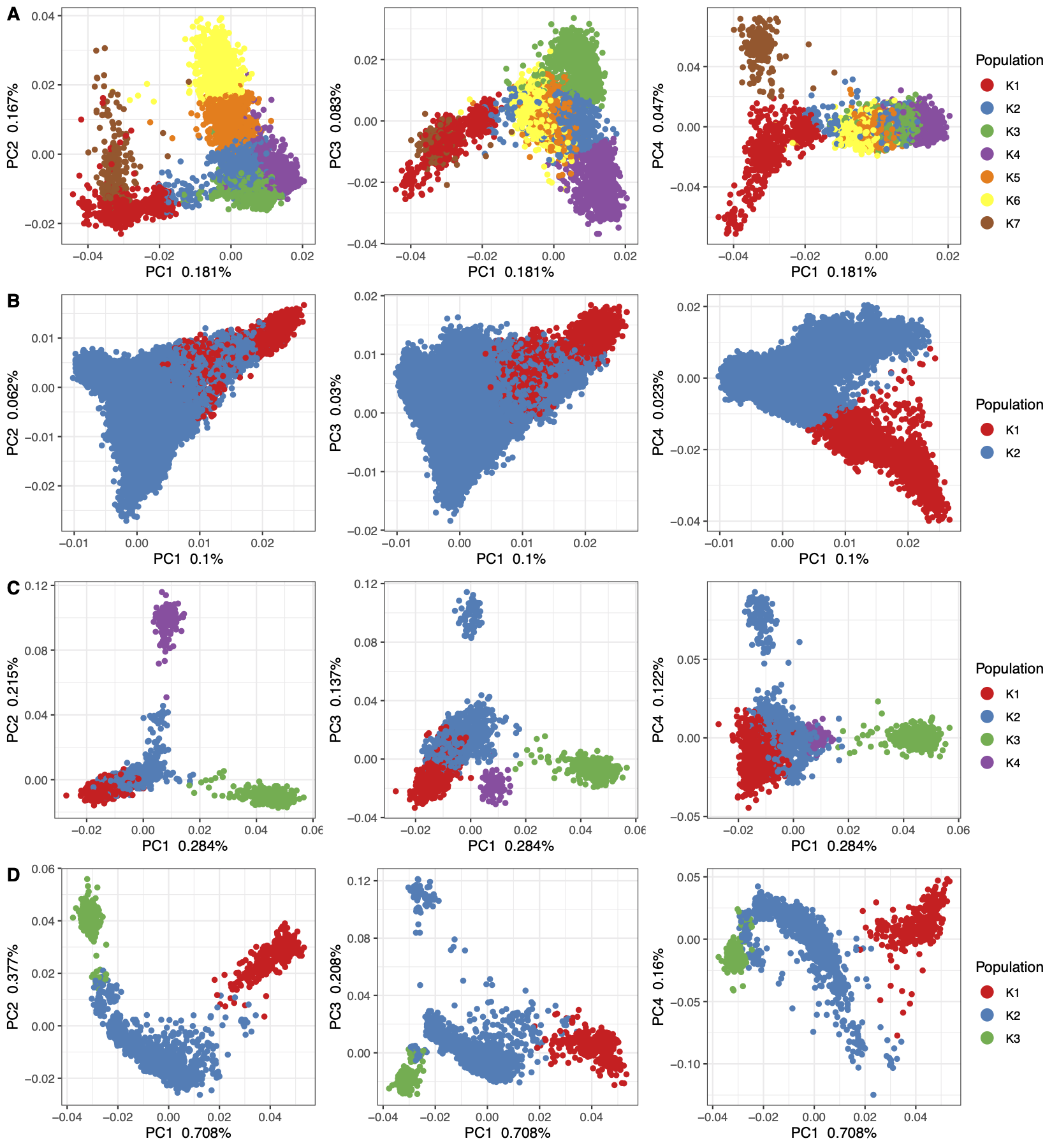


Legend: UK Biobank continental ancestry group PCs 1-4 with k-means groups colour coded: AFR (A), EUR (B), SAS (C), EAS (D).

## Supplementary Figure 4: Population structure by UN defined geographic region.


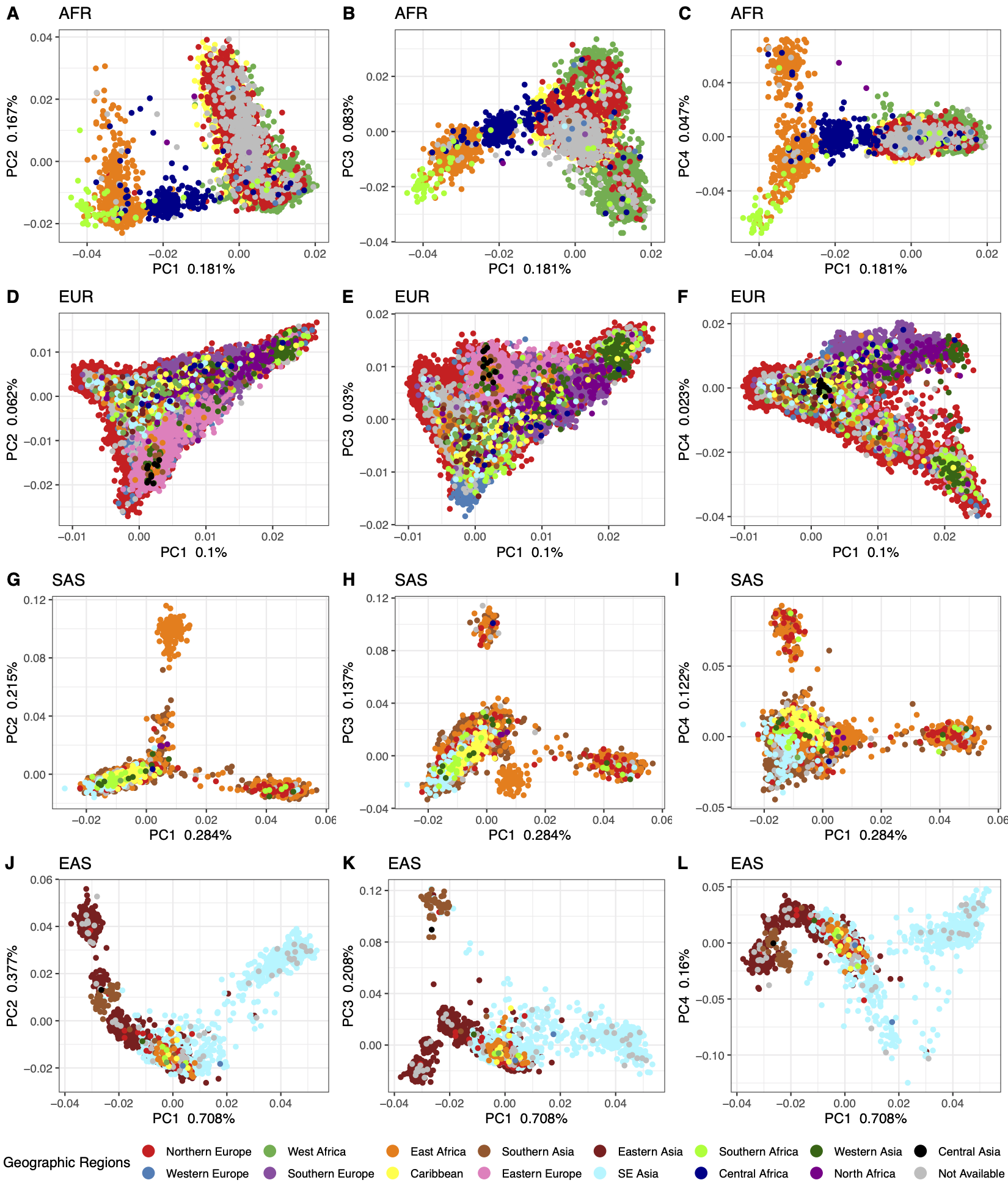


Legend: UK Biobank continental ancestry group PCs 1-4 with regions of birth colour coded: AFR (A), EUR (B), SAS (C), EAS (D).

## Supplementary Figure 5: Population structure centers, as defined by UN geographic region


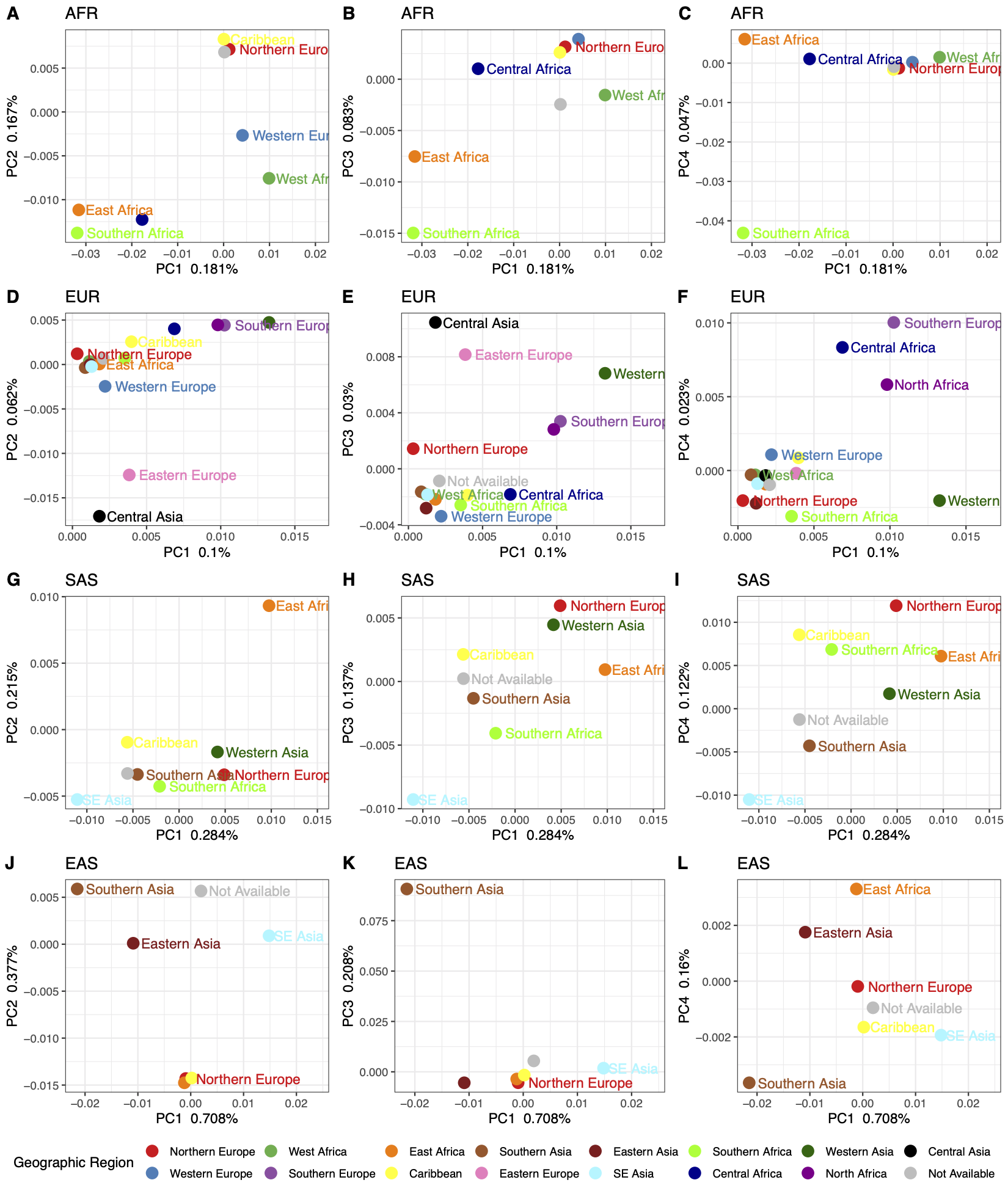


Legend: UK Biobank continental ancestry group PCs 1-4 with region of birth centers (averaged across all individuals from each ROB) colour coded: AFR (A-C), EUR (D-F), SAS (G-I), EAS (J-L).

## Supplementary Figure 6: Population structure by country of birth in AFR by region


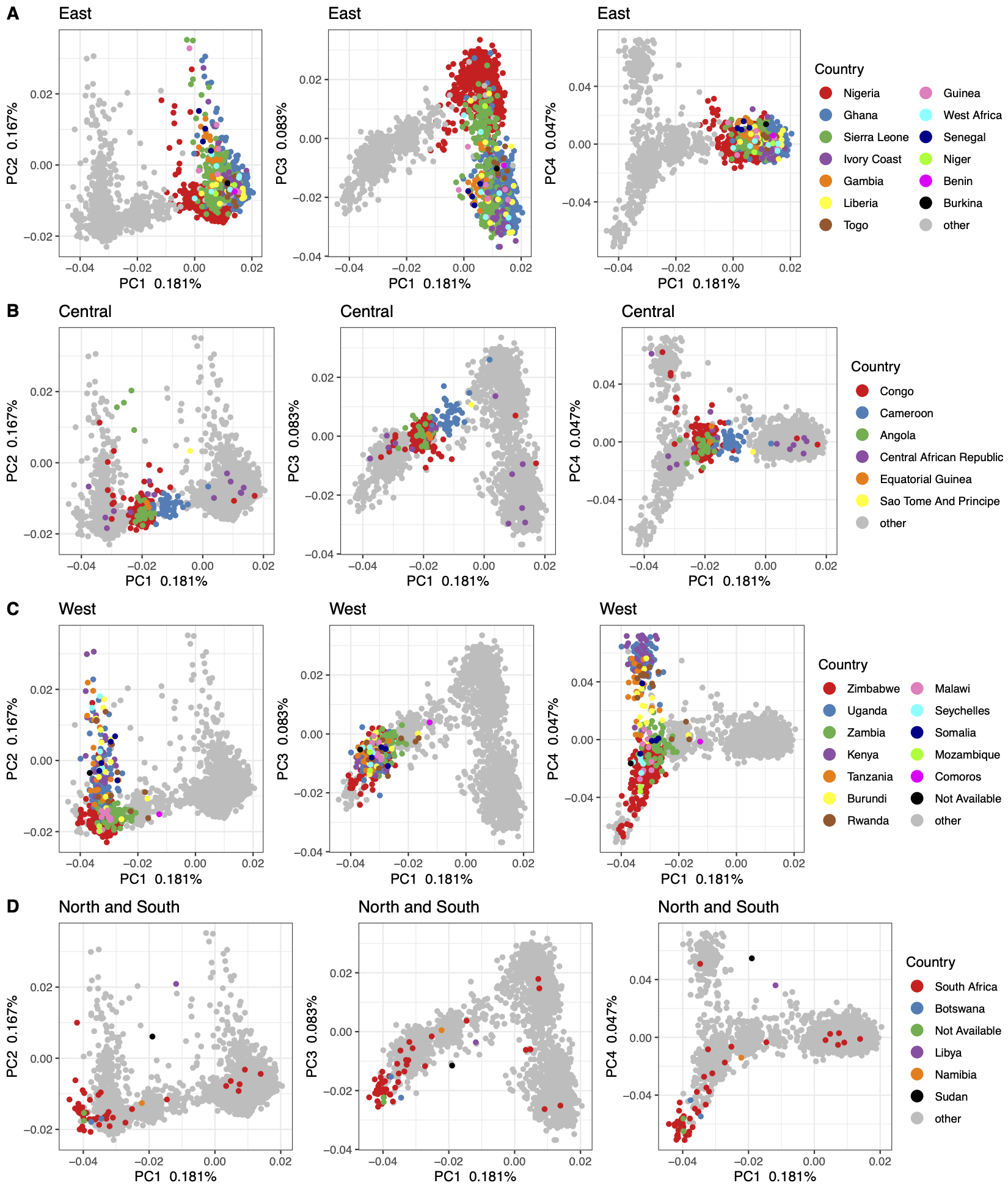


Legend: UK Biobank continental ancestry group PCs 1-4 for the AFR CAG divided by UN regions of birth: Eastern (A), Central (B), Western (C), Northern/Southern (D). Samples are colour coded by their country of birth.

## Supplementary Figure 7: Population structure by country of birth in EUR by region

##
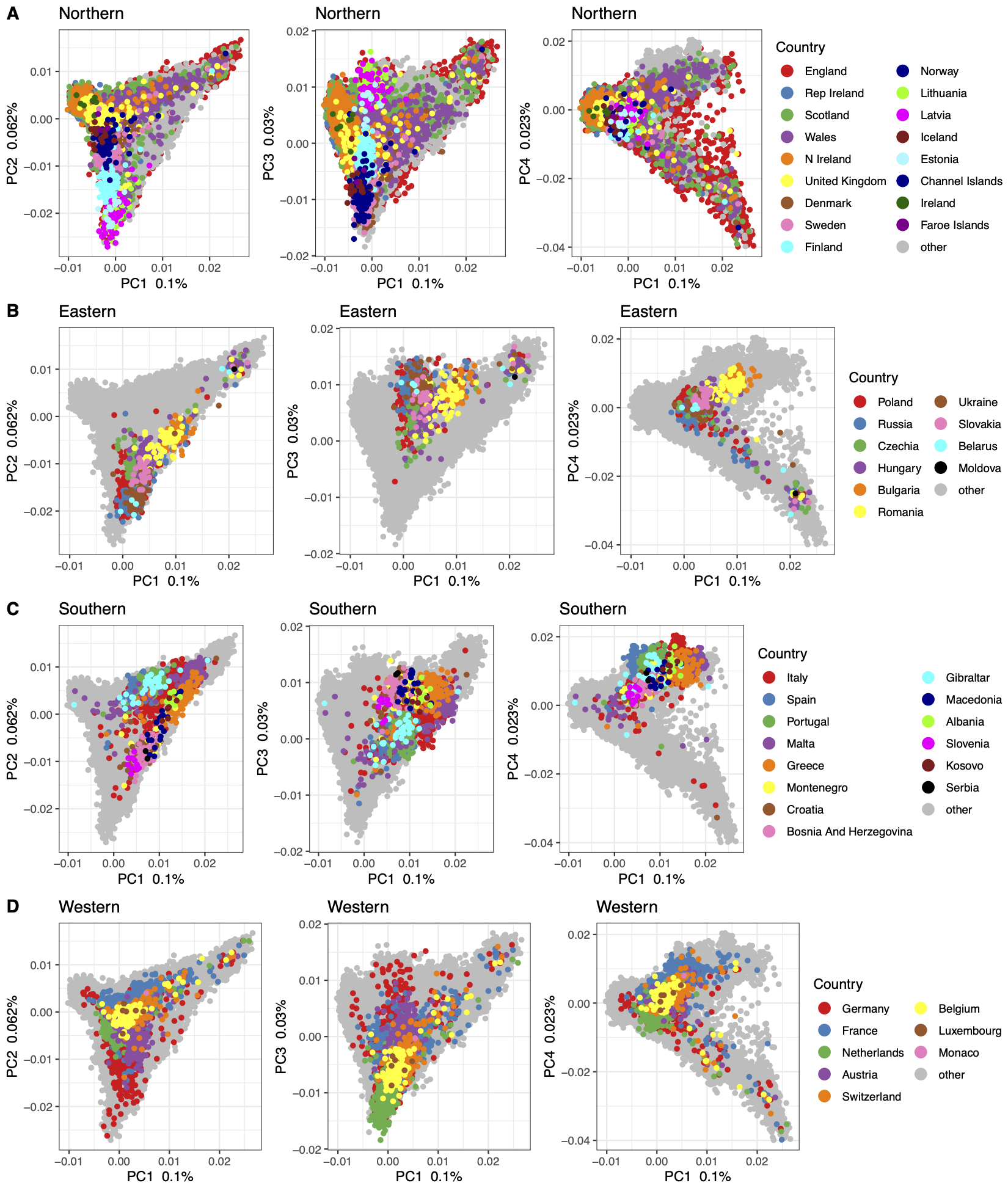


Legend: UK Biobank continental ancestry group PCs 1-4 for the EUR CAG divided by UN regions of birth: Northern (A), Eastern (B), Southern (C), Western (D). Samples are colour coded by their country of birth.

## Supplementary Figure 8: Population structure by country of birth in SAS and EAS


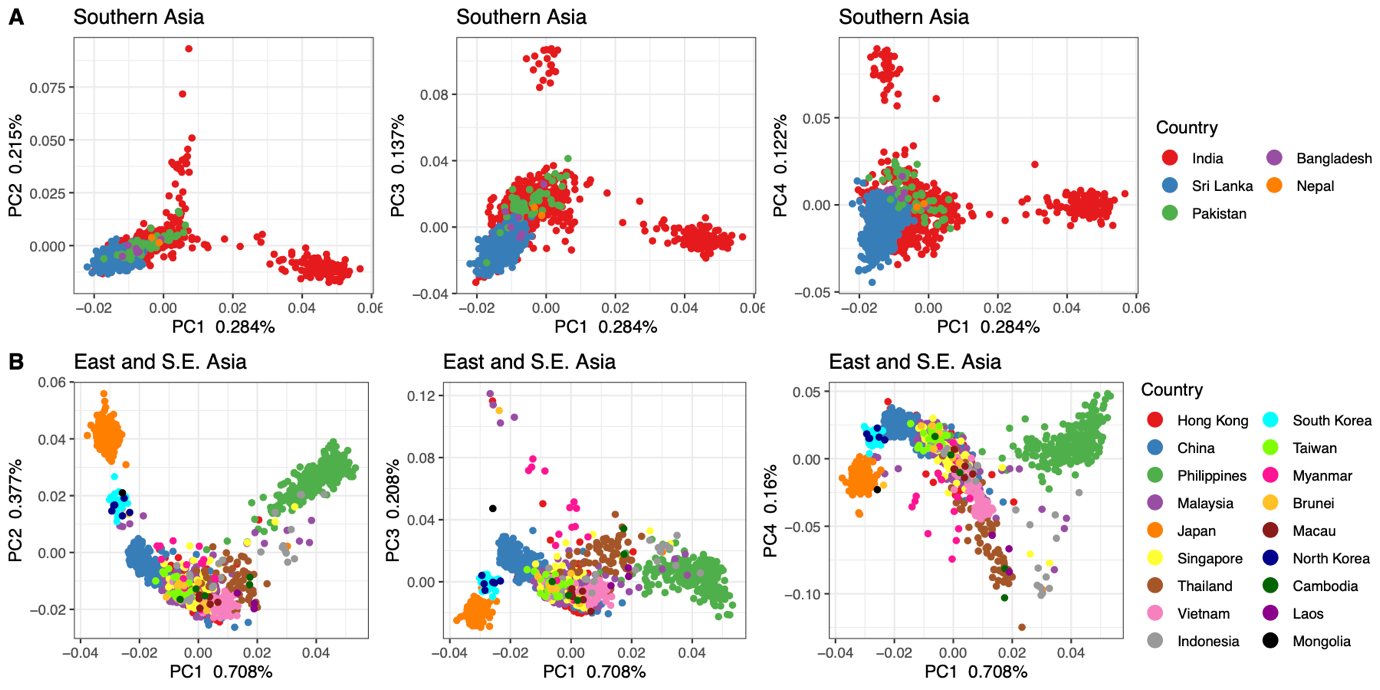


Legend: UK Biobank continental ancestry group PCs 1-4 for the SAS and EAS CAGs divided by UN regions of birth: Southern Asia (A), East Asia and South-East Asia (B). Samples are colour coded by their country of birth.

## Supplementary Figure 9: Population structure centers by country of birth


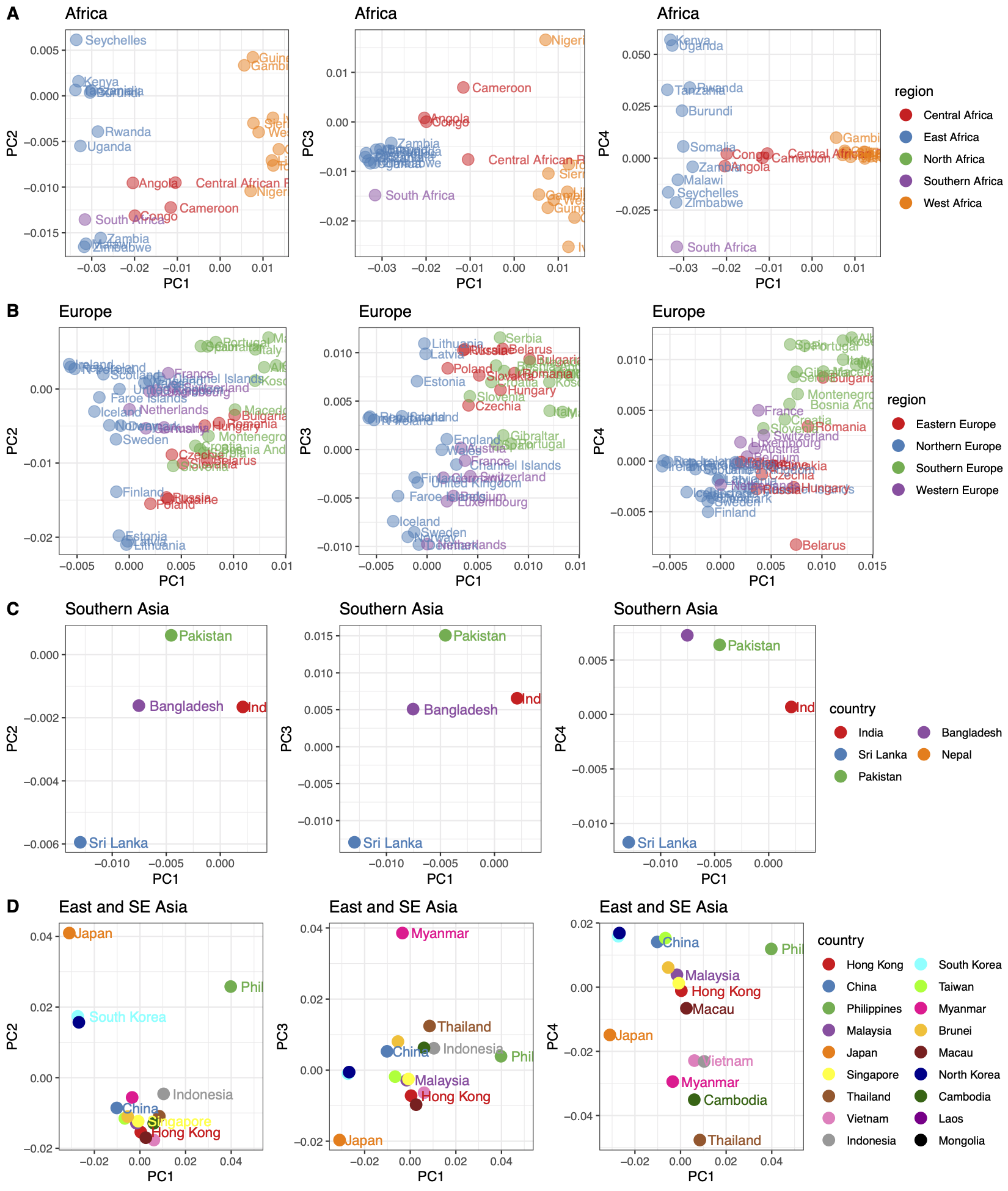


Legend: UK Biobank continental ancestry group centers colored by the country of birth: AFR (A-C), EUR (D-F), SAS (G-I), EAS (J-L).

## Supplementary Figure 10: Population structure centers by country of birth


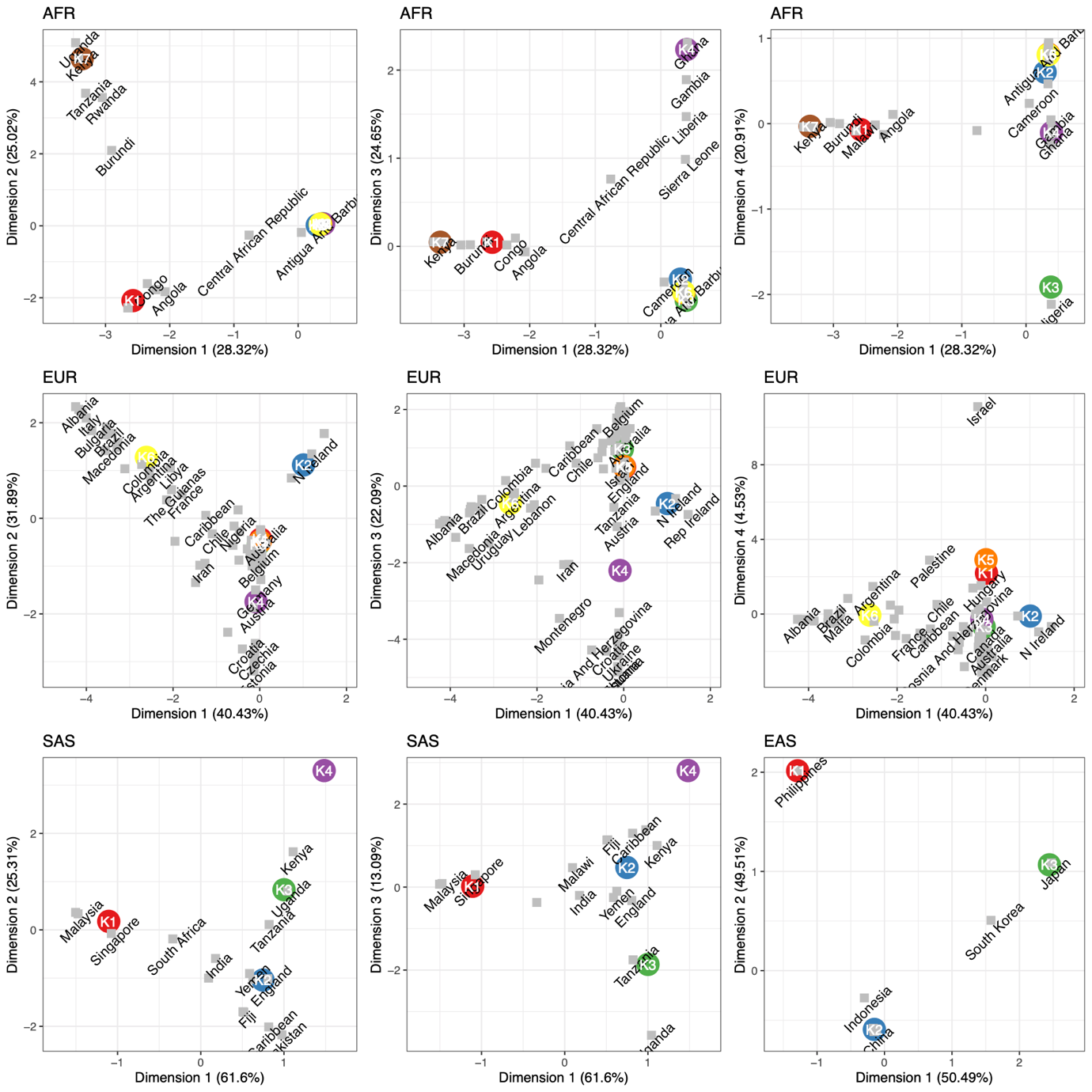
 Legend: UK Biobank continental ancestry group centers in the correspondence analysis, coloured by k-means groups, overlapping with country of birth data in grey: AFR (A-C), EUR (D-F), SAS (G-I), EAS (J-L).
